# Enhancing cold resistance in Banana (*Musa* spp.) through EMS-induced mutagenesis, L-Hyp pressure selection: phenotypic alterations, biomass composition, and transcriptomic insights

**DOI:** 10.1186/s12870-024-04775-5

**Published:** 2024-02-09

**Authors:** Yumeng Liu, Yujia Li, Anbang Wang, Zhuye Xu, Chunfang Li, Zuo Wang, Borui Guo, Yan Chen, Fenling Tang, Jingyang Li

**Affiliations:** 1 National Key Laboratory for Tropical Crop Breeding, Tropical Crops Genetic Resources Institute, Chinese Academy of Tropical Agricultural Sciences, Sanya/Haikou, Hainan 572024/571101 China; 2 Hainan Banana Healthy Seedling Propagation Engineering Research Center, Haikou Experimental Station, Chinese Academy of Tropical Agricultural Sciences, Haikou, 571101 Hainan China; 3 College of Plant Science & Technology, Huazhong Agricultural University, Wuhan, 430070 China; 4 College of Horticulture, Hainan University, Haikou, 571101 Hainan China; 5 Institute of Tropical Bioscience and Biotechnology, Chinese Academy of Tropical Agricultural Sciences, Haikou, 571101 Hainan China; 6 Collage of Tropical Crop, Yunnan Agricultural University, Puer, 611101 Yunnan China

**Keywords:** Bananas, Cold resistance, Somatic mutations, EMS mutagenesis, L-Hyp pressure selection, Transcriptome analysis, Phenotypic variations

## Abstract

**Background:**

The cultivation of bananas encounters substantial obstacles, particularly due to the detrimental effects of cold stress on their growth and productivity. A potential remedy that has gained attention is the utilization of ethyl mesylate (EMS)-induced mutagenesis technology, which enables the creation of a genetically varied group of banana mutants. This complex procedure entails subjecting the mutants to further stress screening utilizing L-Hyp in order to identify those exhibiting improved resistance to cold. This study conducted a comprehensive optimization of the screening conditions for EMS mutagenesis and L-Hyp, resulting in the identification of the mutant *cm784*, which exhibited remarkable cold resistance. Subsequent investigations further elucidated the physiological and transcriptomic responses of *cm784* to low-temperature stress.

**Results:**

EMS mutagenesis had a substantial effect on banana seedlings, resulting in modifications in shoot and root traits, wherein a majority of seedlings exhibited delayed differentiation and limited elongation. Notably, mutant leaves displayed altered biomass composition, with starch content exhibiting the most pronounced variation. The application of L-Hyp pressure selection aided in the identification of cold-resistant mutants among seedling-lethal phenotypes. The mutant *cm784* demonstrated enhanced cold resistance, as evidenced by improved survival rates and reduced symptoms of chilling injury. Physiological analyses demonstrated heightened activities of antioxidant enzymes and increased proline production in *cm784* when subjected to cold stress. Transcriptome analysis unveiled 946 genes that were differentially expressed in *cm784*, with a notable enrichment in categories related to ‘Carbohydrate transport and metabolism’ and ‘Secondary metabolites biosynthesis, transport, and catabolism’.

**Conclusion:**

The present findings provide insights into the molecular mechanisms that contribute to the heightened cold resistance observed in banana mutants. These mechanisms encompass enhanced carbohydrate metabolism and secondary metabolite biosynthesis, thereby emphasizing the adaptive strategies employed to mitigate the detrimental effects induced by cold stress.

**Supplementary Information:**

The online version contains supplementary material available at 10.1186/s12870-024-04775-5.

## Introduction

The adverse impact of low temperatures on bananas, a crop indigenous to tropical and subtropical regions, is well recognized, leading to compromised quality, reduced yield, and even complete crop failure. Consequently, the banana industry faces substantial economic losses. In light of this, the strategic selection of banana cultivars demonstrating resistance to low temperatures becomes paramount to ensuring the sustainable growth of the banana sector. However, the intrinsic characteristics of most banana species, such as polyploidy and parthenocarpy [1], render conventional crossbreeding methodologies unfeasible. Historically, the primary mode of commercial banana propagation has relied on asexual reproduction, which has resulted in the emergence of somatic mutations. While some of these mutations have been deliberately integrated into cultivated varieties through meticulous domestication efforts, enhancing industrial cultivation types [2], spontaneous mutations in naturally propagated bananas remain infrequent. In this context, mutation breeding techniques have proven effective in generating new strains in both seed-bearing and asexual crops [3]. An efficient strategy for generating novel banana cultivars involves mutagenesis combined with somatic embryo regeneration [4–6]. However, it is crucial to note that physical or chemical mutagenesis approaches can lead to elevated mutation rates, potentially resulting in an accumulation of deleterious mutations and a decline in overall fitness levels [7] . Often, detrimental mutations outweigh advantageous ones [8], prompting selective pressure that favors maintaining low mutation rates [9]. Given these considerations, the integration of mutagenesis techniques with stress screening protocols to yield progeny exhibiting desired traits emerges as a more effective and reliable strategy compared to conventional breeding methods. This innovative approach has been instrumental in enhancing favorable attributes of banana varieties by selectively identifying strains demonstrating unique characteristics.

The induction of mutations can be achieved through various methods, including the utilization of transposons and biological reagents such as T-DNA, as well as radiation with physical agents such as ultraviolet rays, X-rays, gamma rays, and fast neutrons [10], and chemical mutagens like diethyl sulfate (DES) or ethyl methane sulfonate (EMS) [11, 12]. These mutagens elicit disturbances in the cellular nucleus’s DNA, resulting in genetic mutations during the process of DNA repair in a stochastic manner. Furthermore, these alterations can also manifest within the cytoplasm, giving rise to chromosomal abnormalities. By selecting advantageous mutations, such as improved flower color [13] and shape [14], disease resistance, and early flowering phenotypes [15], plant breeders can harness the benefits of mutagenesis. It’s important to note that T-DNA and transposon mutagenesis often yield more harmful mutations, while EMS mutagenesis generates different types of mutations within each gene, such as nonsense mutations, missense mutations, splice mutations, and cis-regulatory changes. EMS is also more likely to result in high-density mutations with random distributions [16]. Especially for asexually propagated plants, mutagenic breeding offers advantages due to their cellular totipotency. This totipotency allows the regeneration of complete plants from individual plant cells or tissue cultures. Continuous subculture can further mitigate the emergence of progeny with complex genotypes [17]. Notably, a study on banana meristem shoots treated with EMS demonstrated a high density of GC-AT mutants after six rounds of proliferative multi-cultures, with the chimeric region in EMS-treated meristem tissues rapidly disappearing [18]. These findings have practical implications for mutagenesis-based functional genomics and breeding programs reliant on asexual propagation.

As a general rule, most mutations introduced through random mutagenesis techniques result in non-functional proteins [19]. Consequently, a mutation inhibitor screen is often used to identify mutations that enhance or suppress phenotypes in mutants. To accomplish this, exogenous selection pressure reagents are added to eliminate targets of unexpected traits through directional breeding. Proline, playing a pivotal role in osmotic adjustment in stressed plants, acts as an osmolyte, enabling more effective stress coping by adjusting osmotic potential [20], and Proline likely induces asymmetric induction through iminium ions in plants [21]. Additionally, exogenous proline can enhance stress resistance in plants by acting as a cryo-protectant or osmoprotectant, aiding in cellular osmotic regulation, detoxification of ROS, maintenance of membrane integrity, and stabilization of enzymes and proteins [22]. Both animals and plants produce 4-hydroxyproline (L-Hyp) through the posttranslational modification of proline residues by proline hydroxylase [23]. In the realm of plant biology, L-Hyp exerts its influence by impeding the activity of glutamate kinase, a pivotal enzyme involved in the synthesis of proline. Additionally, it competes with proline for the binding sites on this enzyme. The utilization of L-Hyp in the identification of mutants displaying strong resistance holds notable benefits, primarily due to the substantial buildup of proline within plant tissues during periods of stress. L-Hyp [24]. However, the mechanisms underlying how Hyp induces directional differentiation in vitro for selecting stress-resistant cells remain unclear despite ongoing research in the field.

In our study, we employed in vitro mutagenesis using EMS to generate a diverse population of mutants from *Baxijiao* (*Musa* Cavendish subgroup). Employing a directed screening system using L-Hyp-containing medium, we identified mutants with distinct phenotypic characteristics, including variations in leaf color, shape, and plant type. The dwarf/semidwarf mutants exhibited the highest mutation rates. Subsequent stress rescreening revealed significant improvements in the mutants, particularly in a promising dwarf mutant (*cm784*) exhibiting enhanced low-temperature resistance. This mutant displayed increased proline content, reduced MDA content, and elevated peroxidase and catalase activity, highlighting its superior cold stress tolerance. Additionally, transcriptome sequencing unveiled the molecular basis of cold tolerance in banana mutants. Our study underscores the potential of in vitro mutagenesis and directed screening systems for developing stress-resistant plants. These findings provide valuable insights into the genetic basis and molecular mechanisms underpinning phenotypic diversity and environmental adaptability in banana breeding, offering a foundation for further investigations in this field.

## Results

### Impacts of EMS-induced mutagenesis on growth and development of *Baxijiao* seedlings

The equilibrium between damage minimization and optimal complexity is essential in biological systems [25]. To achieve desired mutants, precise dosages and treatment durations of EMS-induced mutagenesis were critical. Our findings underscored the significant influence of EMS concentration and treatment time on the lethality and differentiation rate of proliferating shoots (Fig. S1). Notably, lethality rates reached 93.94% for the combination of 2.0% EMS + 4 h, highlighting the critical interplay between EMS dose and treatment time. Differentiation rates and multiplication coefficients gradually declined with higher EMS concentrations and longer treatment times. At 2.0% EMS concentration and 4 h treatment, differentiation rate was 0.76%, and multiplication coefficient was 0.045, indicating inhibitory effects on shoot differentiation and growth. However, no significant differences were observed between 1.0% EMS + 4 h and 1.5% EMS + 3 h regarding lethality, differentiation rate, and proliferation coefficient.

**Fig. 1:**
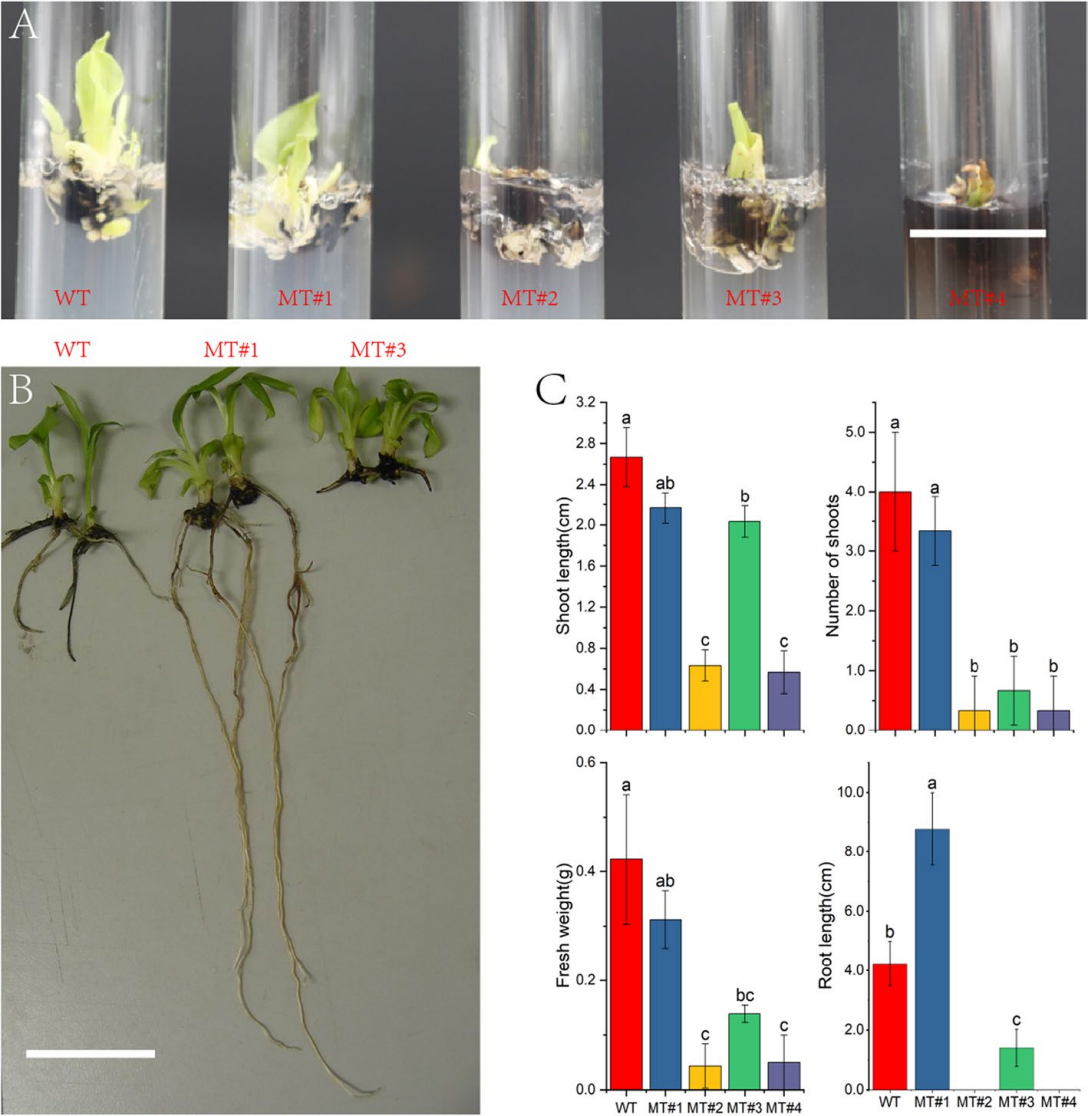
Comparison of wild-type (WT) and EMS-mutagenized bananas (MT) under different growth conditions. **A** The morphology of wild-type and EMS-mutagenized bananas under multiplication conditions after 6 days, (Scale bar = 2 cm), **B** The morphology of wild-type and EMS-mutagenized bananas under rooting conditions after 30 days (Scale bar = 5 cm), **C** EMS concentration and treatment time on shoot length, number of shoots, fresh weight, and root length of mutagenized *Baxijiao* seedlings d*uring* in vitro culture*.* Bars (mean ± SD) that do not share a letter represent significantly different values at *P*<0.05 level (Turkey multiple comparison test)

EMS mutagenesis exerted substantial effects on *Baxijiao* seedlings’ growth and development (Fig. 1A), inducing significant changes in shoot and root characteristics (Fig. 1B and Fig. S2). Quantitative in vitro analyses highlighted differences in shoot length, shoot number, fresh weight, and root length between wild-type (WT) and EMS-mutagenized bananas (Fig. 1C). These variations underscore the significant impact of EMS mutagenesis on shaping the morphology and growth of banana seedlings. Notably, a majority of seedlings exhibited delayed differentiation, hindered root development, and restricted elongation, with a fraction failing to survive the rooting phase. Simultaneously, it was observed that a minute number of root systems experienced growth promotion.

### Interrelated phenotypic variations and traits alterations in EMS-treated banana mutants

**Fig. 2:**
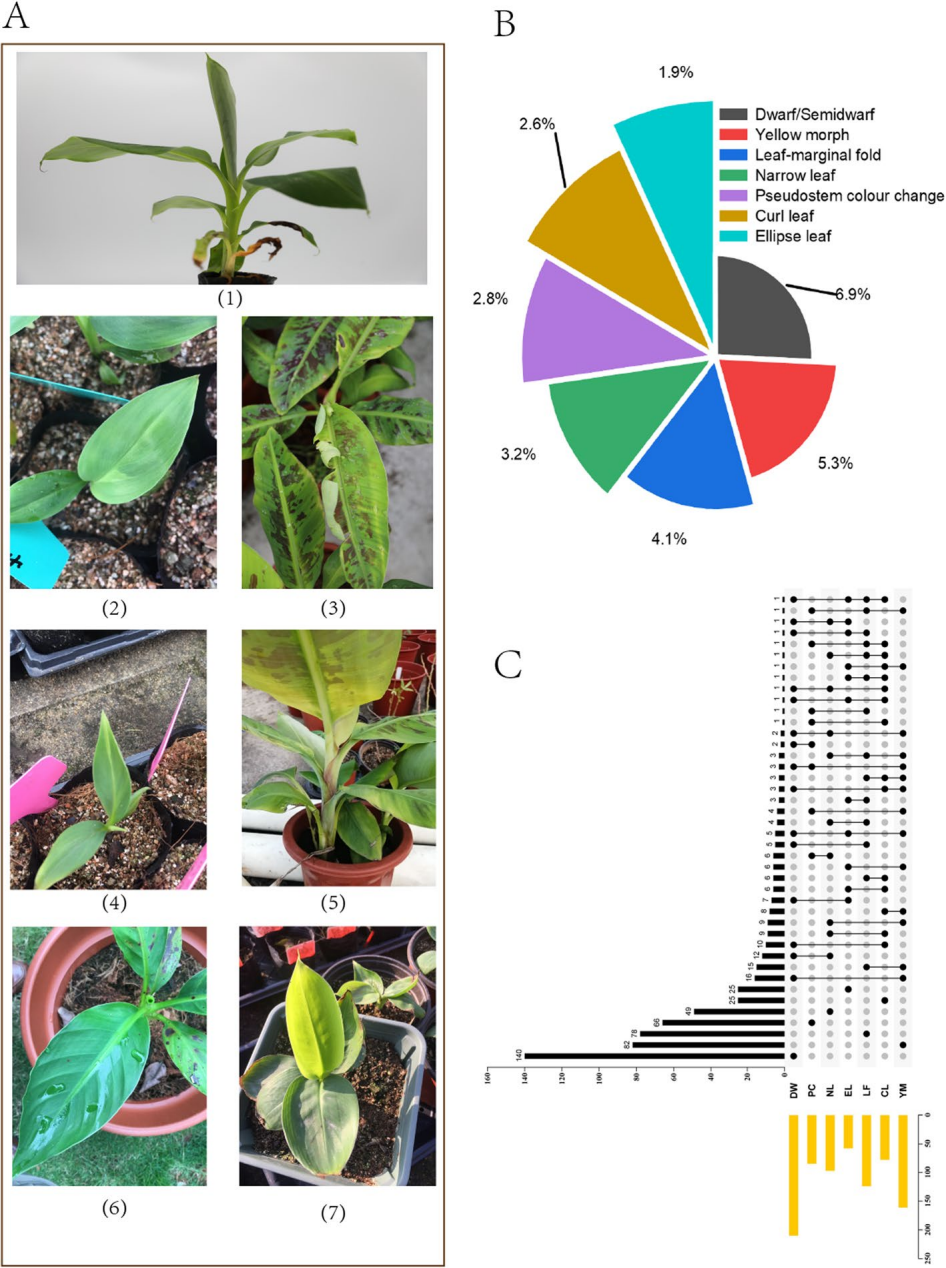
Treatments with EMS/L-Hyp enriched genetic diversity. **A** Different phenotypic variations represented by (1) Dwarf/Semidwarf, (2) Yellow morph, (3) Leaf-marginal fold, (4) Narrow leaf, (5) Pseudostem color change, (6) Curl leaf, and (7) Ellipse leaf. **B** The percentages represent the occurrence of different mutant phenotypes. **C** Upset plot of banana phenotype mutants. Upset plot showing the overlap of off-types in the phenotype of mutated *Baxijiao* progeny(*n* = 787). DW, Dwarf/simi-dwarf; PC, Pseudo-stem color change; NL, Narrow leaf; EL, Ellipse leaf; LF, Leaf-marginal fold; CL, Curl leaf; YM, Yellow morph

During acclimatization, mutant banana plants displayed diverse phenotypic variations, including leaf color, type, and plant structure mutations. Notably, 6.9% of *Baxijiao* plants exhibited dwarf or semi-dwarf traits, with some showing extreme shortness. Mutant leaf types showed features such as folded margins, curling, and narrow elongated forms (Fig. 2A), at rates of 4.1, 2.6, and 3.2% respectively. Rates of leaf yellowing, pseudo stem color change, and elliptical leaves were 5.3, 2.8, and 1.9% respectively (Fig. 2B). In tissue culture seedlings, distinctive phenotypic traits such as dwarfism, narrow leaves, and leaf curl were markedly more pronounced. This heightened manifestation of mutant phenotypes in the controlled environment of tissue culture suggests a potent influence of growth conditions on the expression of EMS-induced mutations. Intriguingly, as the plants progressed through subsequent growth stages and entered the acclimation phase, there was a gradual attenuation of these initially prominent mutant characteristics. This observation implies a dynamic interplay between EMS mutation effects and the evolving growth environment, possibly involving physiological repair mechanisms that mitigate the impact over time.

Our analysis of 532 mutants showed that the majority (92.9%) exhibited 1–2 genetic alterations in phenotype (Fig. 2C). Categorizing changes into distinct groups based on main characteristics revealed that most mutants-maintained phenotype stability across various seasons. These findings suggest that phenotypic variations in bananas primarily stem from genetic factors, supported by high broad-sense heritability estimates [26]. Implying significant implications for banana breeding, these results underscore the potential for genetic manipulation to enhance key agronomic traits in this vital crop.

### Altered biomass composition in banana mutants

**Fig. 3:**
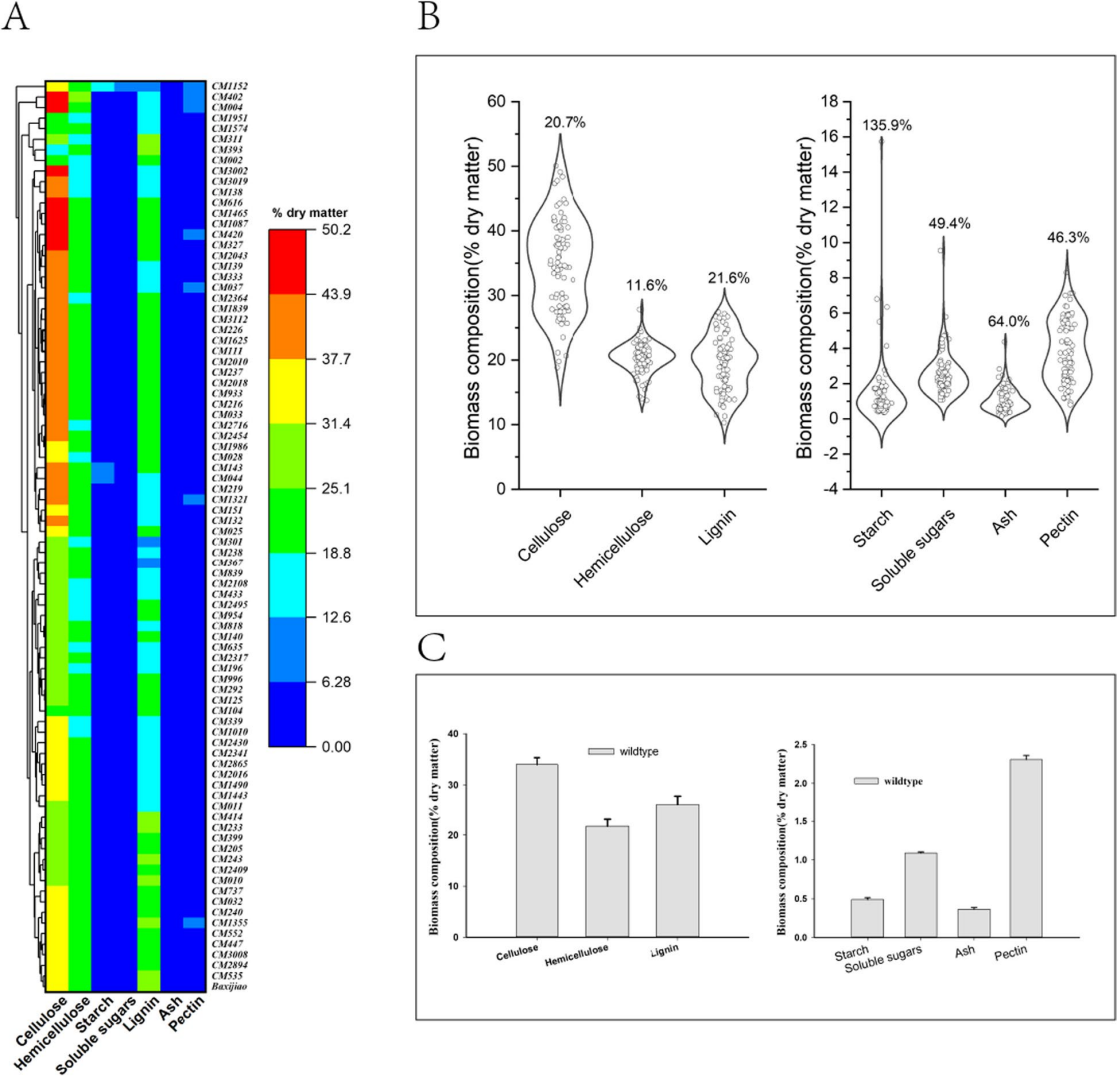
Treatments with EMS/L-Hyp altered banana biomass composition and enriched genetic diversity. **A** Cluster heatmap of biomass composition of mutagenized banana leaf. **B** Violin plot represents data density(*n* = 82), the upper value of the plot represents %CV. **C** The mean value of biomass for the wild type (*Baxijiao*)

We further investigated the impact of phenotypic changes on the composition of banana mutant biomass, specifically focusing on soluble sugars, starch, cellulose, hemicellulose, lignin, and pectin. In total, 81 mutants and the wild type were assessed, revealing that EMS mutagenesis significantly altered the biomass composition of banana mutant leaves, as demonstrated by a cluster heatmap (Fig. 3A). Additionally, a violin plot was utilized to visually depict the distribution of data concerning the coefficient of variation (CV) values among 81 mutants and the wild type in relation to banana leaf biomass content (Fig. 3B and C). These CV values were employed to quantitatively assess the level of diversity in terms of leaf biomass content in bananas. Remarkably, the mutants’ biomass displayed a range of CV values from 11.61 to 135.85%. Notably, the starch content exhibited the highest CV value, underscoring significant compositional variations in banana leaf biomass due to the mutagenesis process. The sensitivity of CV to outliers, particularly evident in sugar content, adds nuance to the observed changes. This phenomenon highlights the substantial influence of mutagenesis on the complex metabolic pathways involved in biomass synthesis in banana plants.

### Enhancing cold-resistant mutant screening with L-Hyp pressure selection

Enhancing breeding efficiency through early selection can effectively mitigate randomness and inefficiencies in mutation breeding [27]. In this study, we developed a genetic screening method to identify cold-resistant mutants among banana mutants displaying seedling-lethal phenotypes. This strategy capitalized on L-Hyp-induced lethal plants to isolate potential cold-resistant mutants. Comparative analysis between wild type (*Baxijiao*) and EMS-mutagenized seedlings revealed that in vitro shoots exhibited an average survival rate of 68.9% at L-Hyp concentrations below 0.6 mmol/L, indicating *Baxijiao*’s tolerance to low L-Hyp levels. However, as L-Hyp concentrations increased, survival rates declined, with only 5.6% of in vitro shoots surviving at 1.2 mmol/L (Fig. S3). Importantly, EMS-mutagenized seedlings displayed higher survival rates than the wild type as L-Hyp concentration exceeded 0.4 mmol/L, suggesting EMS-induced mutations’ potential to enhance survival under elevated L-Hyp concentrations. A similar observation was made in a prior study [28], where selection pressure favored retention of resistant alleles while eliminating sensitivity-associated alleles. Setting selection pressure at 1.0 mmol/L L-Hyp based on EMS-mutagenized seedling survival rates could maximize the identification of mutants resilient to further manipulation.

### Identification of mutant *cm784* through L-Hyp screening of EMS mutagenized progeny

**Fig. 4:**
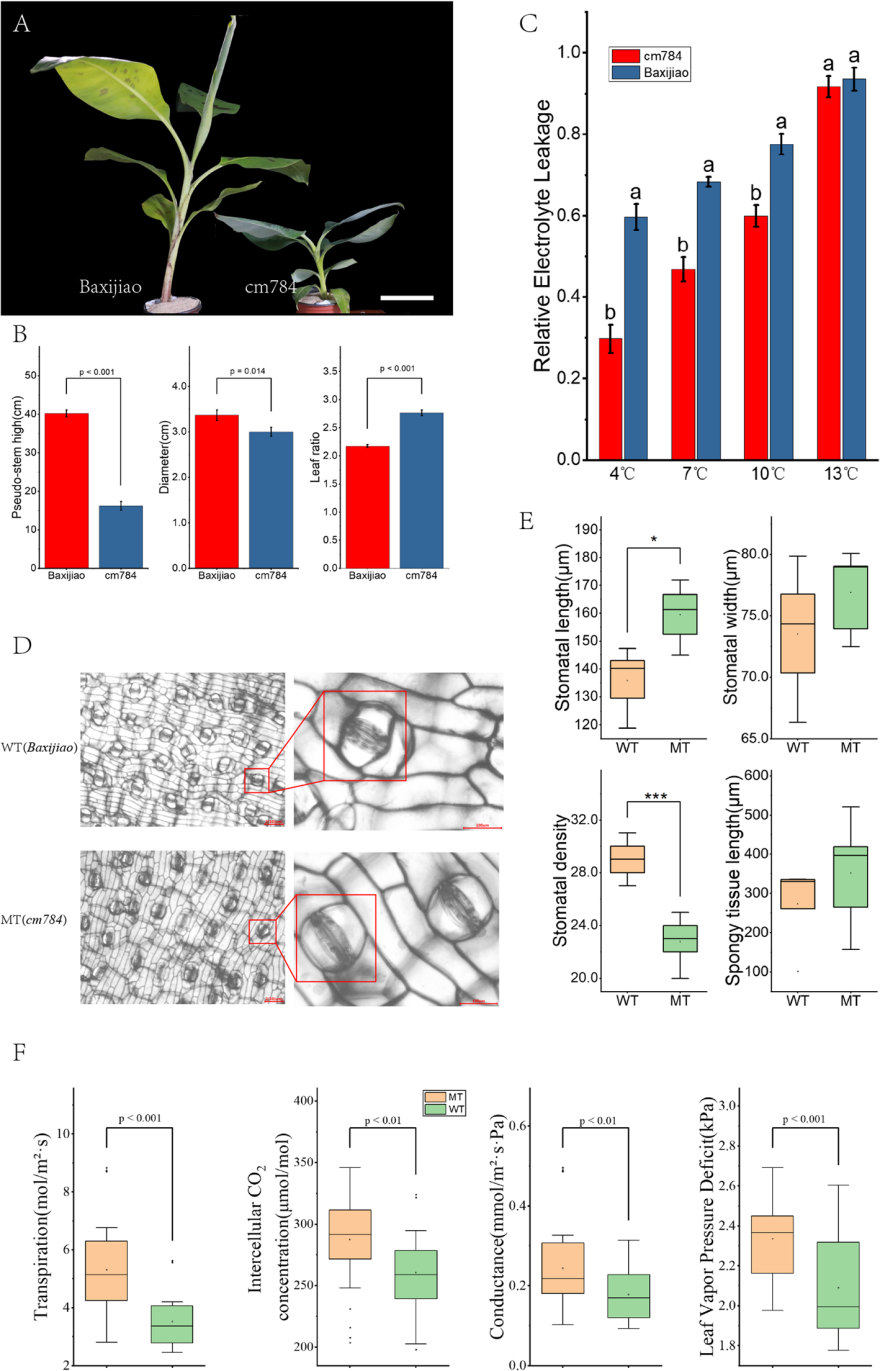
Identification and characterization of the *cm784* mutant. **A** Morphological comparison of *Baxijiao* and *cm784* seedlings. Scale bar = 15 cm. **B** Comparison of Pseudo-stem height, diameter, and Leaf ratio (ratio of the length of the longest leaf to the length of the stem) in seedlings after 45 days of growth in pots. Statistical significance assessed by ANOVA with Turkey’s post hoc test. **C** Conductivity of leaf at various temperatures between B*axijiao* and *cm784*. The same letter indicates no significant difference at *p* < 0.05(Turkey’ s test). **D** Comparison effects of EMS/L-Hyp treatment on the changes of stomatal opening rate between wild type (*Baxijiao*) and mutation type (*cm784*). Leaf samples from both the wild-type (WT) and mutant (MT) plants were observed under a microscope, with each visual field equivalent to 10 μm (Bar scales = 10 μm). Insets showing high-magnification views of the boxed areas are shown on the right (Bar scale = 100 μm). **E** Comparisons of stomatal length; stomatal width; stomatal density and Spongy tissue length between WT and MT. Asterisks indicate significance in paired Tukey test, *P < 0.05, **P < 0.01, ***P < 0.001

After subjecting the EMS-induced population to L-Hyp pressure selection, we successfully identified a mutant named *cm784* during the acclimatization process. After a 45-day period of culture hardening, the seedlings of *cm784* exhibited a noticeable dwarf-like phenotype (Fig. 4A and B) and in contrast to the trend observed and reported by Yang et al. [29], our study reveals that *Baxijiao* and *cm784* exhibit varying levels of relative electrolyte leakage under cold stress at different temperatures. Notably, the cm784 line demonstrates significantly reduced relative electrolyte leakage compared to the wild type, suggesting that the mutations contribute to enhanced cold tolerance. (Fig. 4C). It is important to acknowledge that the development of mutants might have been influenced by mutagenesis, which could explain the observed divergences in growth rate compared to the WT.

We conducted additional experiments to evaluate the effects of mutagenesis on leaf stomata. An analysis of stomatal density, size, and distribution was carried out on leaves from both the wild type (*Baxijiao*) and the mutated type (*cm784*). The captured images provided clear visual evidence of the contrasting stomatal morphologies between the two plant types (Fig. 4D). Subsequent investigations revealed significant effects of mutagenesis on stomatal density and size, exemplified by the larger dimensions of stomata in *cm784* compared to the wild type (WT) (Fig. 4E). Our study findings suggest that *cm784* exhibits a notably heightened stomatal activity in comparison to the wild type. This observation is substantiated by comprehensive tests on photosynthetic efficiency, encompassing conductance, intercellular CO_2_ concentration, transpiration, and leaf vapor pressure deficit (Fig. 4F). Also, these parameters are reduced in the mutant as compared to the wild type, suggesting reduced stomatal activity. These outcomes underscore the intricate interplay among these parameters and the specificity of stomatal function. Collectively, these observations emphasize the discernible impact of mutagenesis on stomatal characteristics, implying potential physiological alterations induced by the mutagenic treatment.

### Physiological responses of *cm784* under low temperature stress

**Fig. 5:**
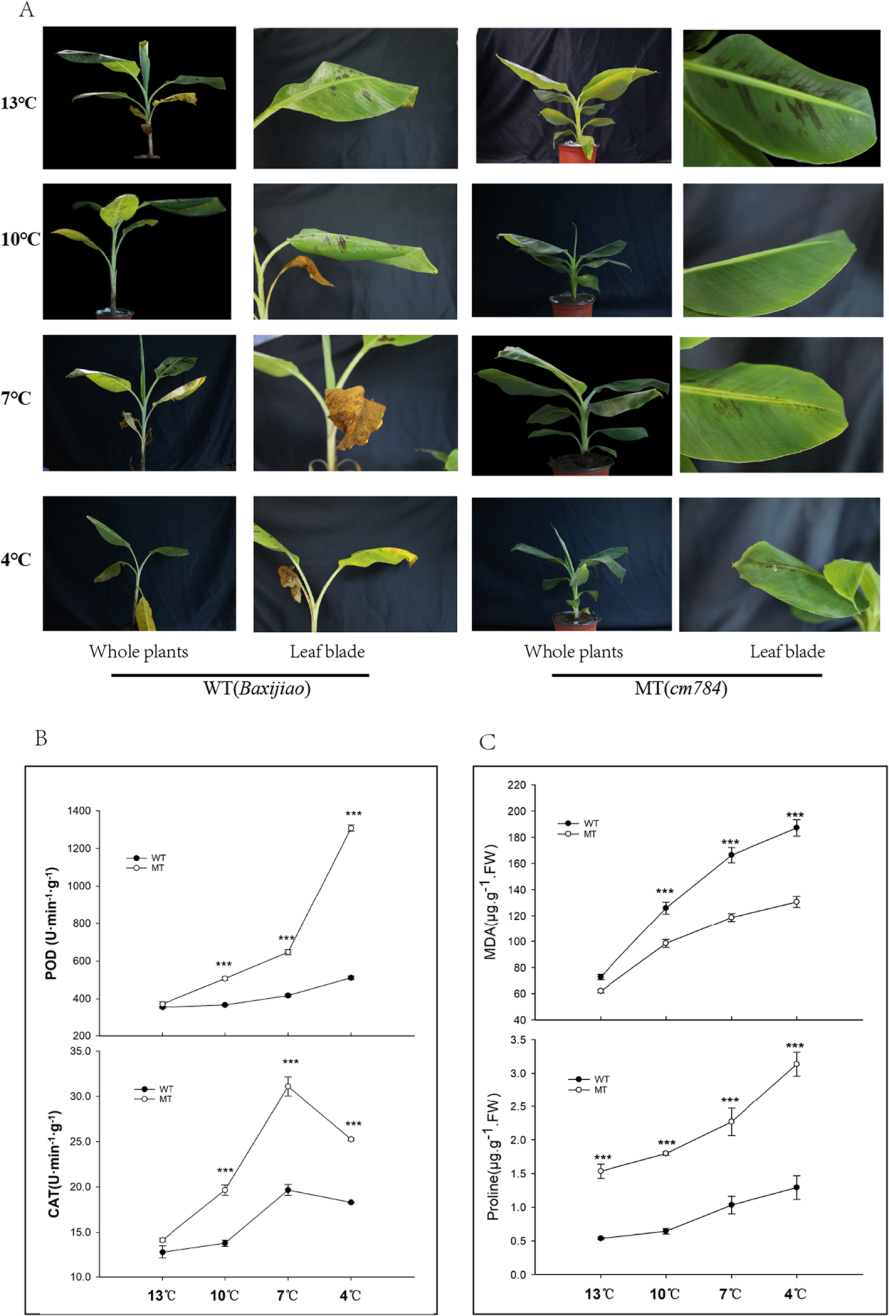
Assessment of seedling chilling injury (CI) index. **A** Chilling injury symptoms of WT (*Baxijiao*) and MT (*cm784*) banana seedlings recovered 1 week after 13 °C,10 °C,7°Cand 4 °C stress, respectively; POD and CAT (**B**), as well as MOD and Proline (**C**) data measured in young leaves of WT and MT during low temperatures. WT, wild-type; MT, mutant-type. Asterisks indicate significance in paired Tukey test, ∗ *p* < 0.05. ∗∗ *p* < 0.01. ∗∗∗ *p* < 0.001

The impact of low temperature stress on the banana mutant *cm784* and the wild type (WT) was investigated in terms of their antioxidant enzyme activities and stress tolerance mechanisms. This study investigated the phenotypic characteristics of *cm784* and WT under varying low-temperature conditions, as well as the alterations in enzyme activity of catalase (CAT) and peroxidase (POD). After subjecting cm784 and WT to temperature treatments ranging from 13 °C to 4 °C for a duration of 48 hours, the extent of freeze damage was assessed. It was observed that at 13 °C, there was no significant disparity in freeze damage between the mutant and wildtype. However, as the temperature decreased, WT displayed more pronounced freeze damage in comparison to *cm784*. Specifically, at 4 °C, WT leaves exhibited a yellowing and drying phenomenon, whereas *cm784* only exhibited mild freeze damage (Fig. 5A). The enzymatic activities of CAT and POD in *cm784* were notably higher compared to WT under the prevailing conditions. As the stress temperature decreased, CAT enzyme activity showed an initial rise followed by a decline. At 7 °C, peak enzyme activity was observed, with *cm784* (31.1 U.min^−1^. g^−1^) displaying a significant 11.72% increase in CAT activity compared to WT (19.7 U.min^−1^. g^−1^). Similarly, POD enzyme activity gradually increased with decreasing temperature, and *cm784* consistently exhibited higher enzymatic activity than WT, the highest POD enzyme activity was observed at 4 °C, with cm784 (1307.3 U.min^−1^. g^−1^) showing a 2.03-fold increase over WT (511.9 U.min^−1^. g^−1^) (Fig. 5B).

Furthermore, to assess oxidative damage, membrane lipid peroxidation was gauged through malondialdehyde (MDA) levels. As temperature decreased, MDA levels rose, yet *cm784* consistently maintained lower MDA content compared to WT. At 4 °C, *cm784* exhibited the highest MDA content (199.14 μg/g) in contrast to WT (148.63 μg/g), implying reduced membrane damage in the mutant (Fig. 5C). Concurrently, proline concentration incrementally increased with decreasing temperature (Fig. 5C), with *cm784* consistently presenting higher proline content than WT. At 4 °C, *cm784* showcased the highest proline content (3.043 μg/g), significantly exceeding WT (1.51 μg/g). These findings unequivocally illustrate that *cm784* displayed heightened antioxidant enzyme activities and proline production when confronted with cold stress, contributing to its elevated cold resistance compared to WT. The results underscore the pivotal role of enzymatic activities and osmoprotectant accumulation in amplifying stress tolerance within banana mutants.

### Enhanced cold resistance mechanisms revealed in mutant banana transcriptome

**Fig. 6:**
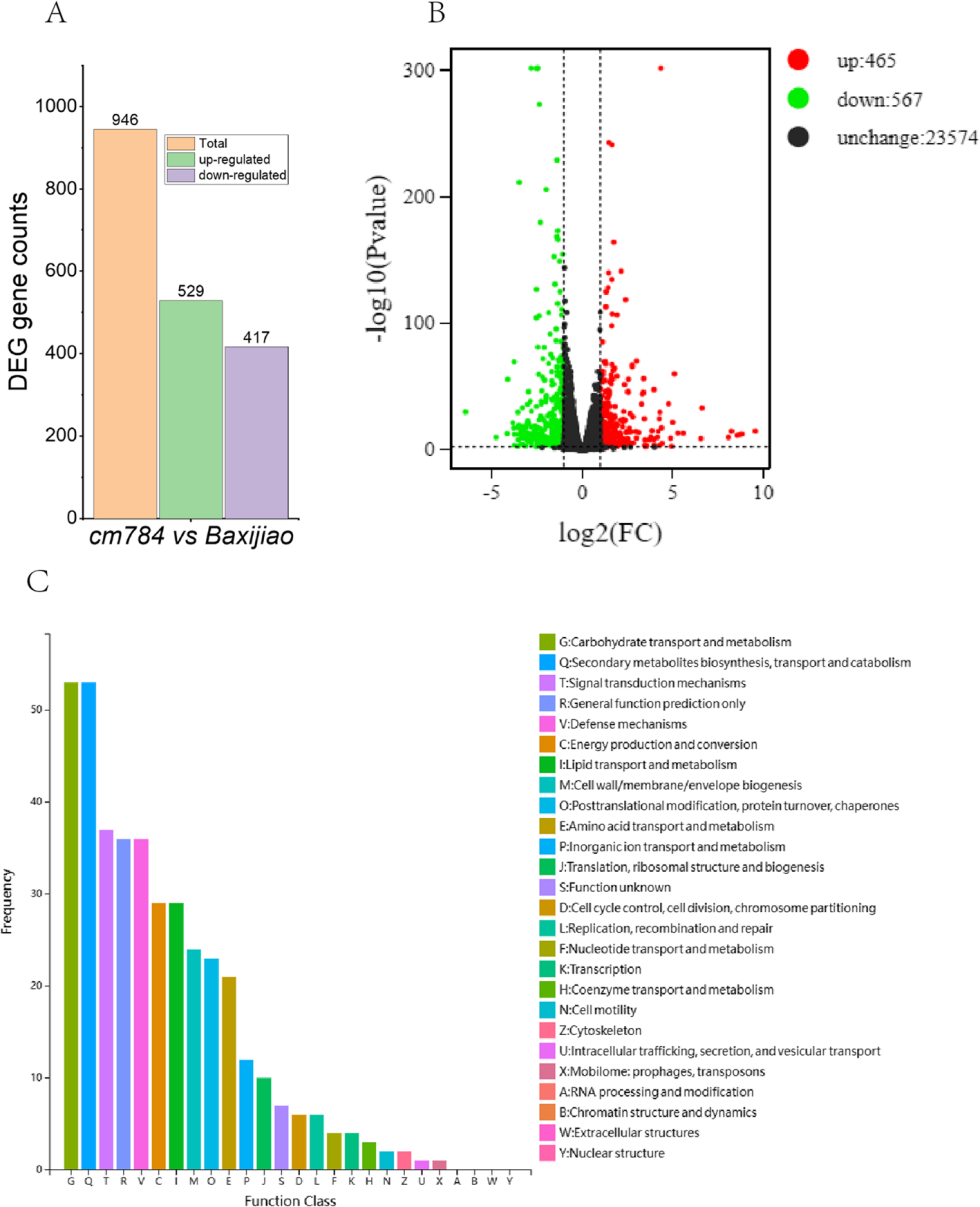
Transcriptome analysis of the *cm784* mutant and wild-type banana (*Baxijiao*). **A** Differentially expressed gene (DEG) analysis. **B** Volcano plot illustrating fold change and statistical significance. Upregulated genes shown in red, downregulated genes in blue, and unchanged genes in black. **C** COG function classification of transcriptomes

To unravel the molecular mechanisms underlying enhanced cold resistance in *cm784*, we conducted transcriptome analysis. Differential gene expression analysis identified 946 differentially expressed genes (DEGs), with 529 upregulated and 417 downregulated in *cm784* compared to wild-type banana (Fig. 6A). A Volcano plot illustrated fold change and statistical significance, revealing 465 upregulated and 567 downregulated genes, while 23,574 remained unchanged (Fig. 6B). Functional categories enriched in the mutant were analyzed using the Cluster of Orthologous Groups of proteins (COG) database. Notably, ‘Carbohydrate transport and metabolism’ and ‘Secondary metabolites biosynthesis, transport, and catabolism’ exhibited the highest frequency values (Fig. 6C).

Furthermore, our study underscores the importance of delving into the observed modifications in the biomass composition of the *cm784* mutant. Particularly, the substantial fluctuations in starch content and its alignment with the transcriptomic profile merit in-depth exploration. This investigation is pivotal for unraveling the molecular mechanisms governing improved cold resistance, shedding light on the role of altered carbohydrate metabolism in the mutant’s adaptive strategies during cold stress. The enrichment of ‘Secondary metabolites biosynthesis, transport, and catabolism’ suggests their crucial involvement in cold tolerance. Higher frequency values imply enhanced biosynthesis, transport, and catabolism of secondary metabolites in the cold-tolerant mutant, fortifying protection against cold-induced damage. By unveiling molecular mechanisms related to carbohydrate transport, metabolism, and secondary metabolite processes, our study provides a comprehensive understanding of the adaptive strategies employed by the mutant banana against cold stress.

## Discussion

### Exploring the unique approach of mutation breeding and the role of chemical mutagens in enhancing mutation frequency

The application of mutagenesis as a breeding method presents a promising avenue for generating novel variants through diverse mutations, encompassing DNA base conversions, substitutions, and deletions. These mutations, arising from both spontaneous and induced processes, serve as valuable sources of new alleles [30] . Notably, our research underscores the significant advantages of mutation breeding, particularly through the strategic use of chemical mutagens, allowing us to surpass mutation frequencies observed in natural processes by over 100-fold. Mutation breeding stands apart from combination breeding and heterosis breeding by relying on distinct sources of genetic variation. Our study highlights the unique traits introduced by mutagenesis, often extending beyond the scope of natural variation, thereby enriching the diversity of selectable traits in breeding programs. The optimization of EMS mutagenesis concentration broadens the mutation spectrum and contributes to enhanced mutation frequency [5]. Induced mutations, with a focus on limited loci, sometimes as few as two, prove ideal for refining existing superior traits through controlled crosses. This controlled approach has proven effective in generating desirable mutant traits such as dwarf stature, early maturity, and disease resistance [31] . These traits, often elusive through natural variation, significantly contribute to the improvement of crop varieties. Particularly crucial is the application of mutagenesis-based breeding to refine established banana varieties that already possess commendable traits but may lack in specific aspects. This endeavor holds substantial scientific importance within the broader context of advancing sustainable agriculture and enhancing global food security.

### Strategic insights into banana mutagenesis

In accordance with a common practice, mutagen doses that lead to approximately 50% lethality in M_1_ plants are generally considered appropriate, given their high mutation rate [32]. To ensure a desirable mutation rate, it is crucial to determine the degree of semi-lethality in samples treated with mutagens at specific doses and durations. Previous studies have made researching EMS-induced mutagenesis feasible [33]. However, it is important to note that different crops exhibit varied sensitivity to EMS mutagens. For instance, in Chinese cabbage, the treatment of buds with EMS concentrations of 0.03 to 0.1% for five to 10 minutes resulted in the highest embryo production and seedling rate, whereas higher concentrations were found to be lethal to microspores [34]. In the case of *Cucurbita pepo* seeds, treating them with 0.3% EMS for 12 hours was identified as the most effective method for inducing mutagenesis. Although the said treatment had no impact on the germination rates of M_1_, it did reduce the fertility of M_1_ plants to 54% as observed in the study conducted by García et al. [35]. The present study established that, in terms of differentiation rate, the optimal condition for EMS mutation of proliferating shoots of ‘*Baxijiao*’ was found to be 1.5% + 3 h, offering an ideal mutation density and dependable M_1_ mutant number.

### Enhancing cold stress resilience through novel L-Hyp-induced screening approach

**Fig. 7:**
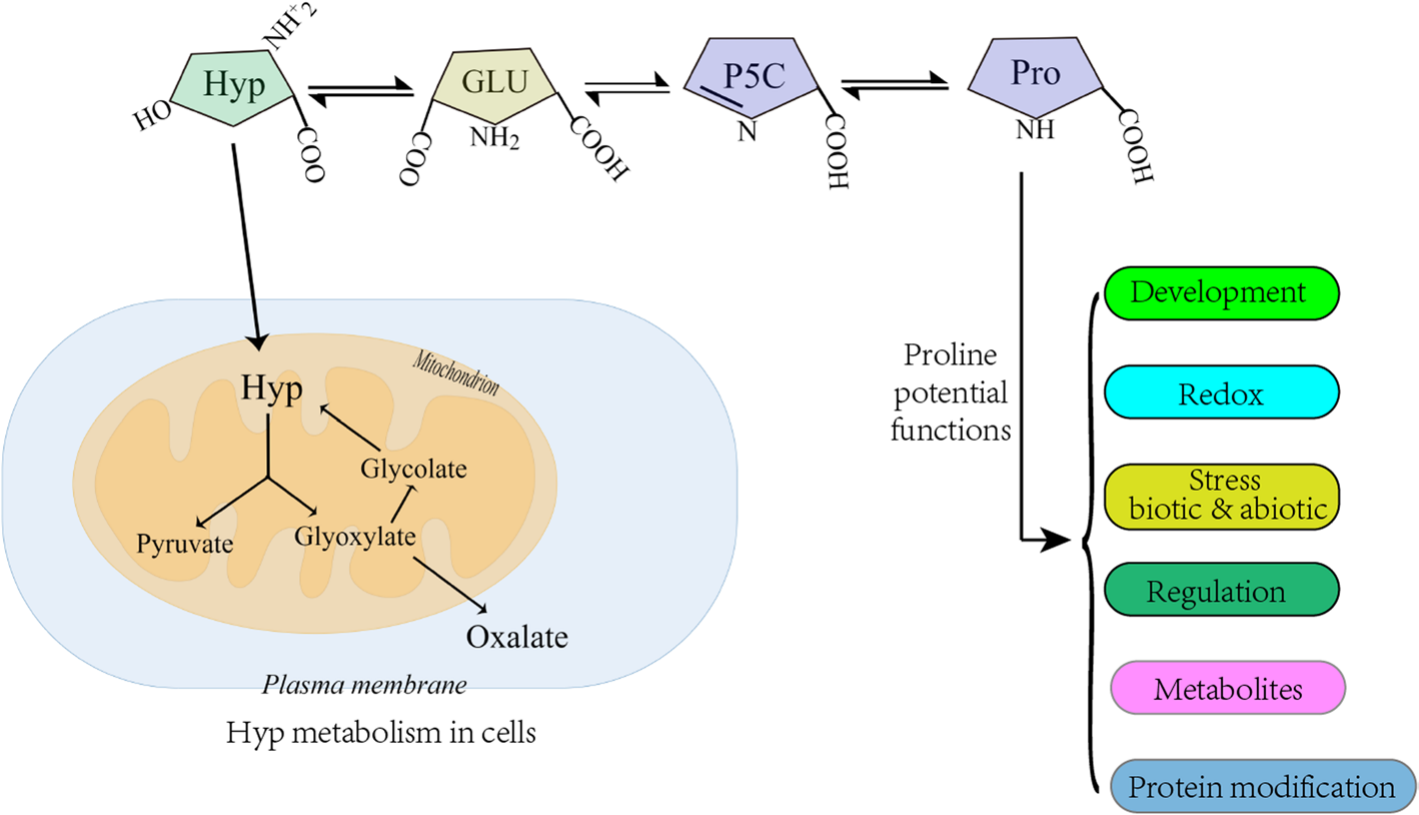
Metabolic pathways and roles of Proline in plant stress responses. Proline plays pivotal roles in various aspects of plant stress adaptation, including development, redox homeostasis, biotic and abiotic stress tolerance, regulation of gene expression, modulation of metabolites, and protein modifications

The metabolic role of proline encompasses a range of complex functions in organisms, including signaling, stress protection, and energy production [36]. Moreover, proline influences development, redox balance, and stress responses against biotic and abiotic factors. Proline acts as an osmoprotectant, stabilizing cellular structures under stress (Fig. 7). Notably, plant tissues are susceptible to damage by Hyp, a proline analog that competes for proline and hampers glutamate kinase, consequently inhibiting proline synthesis [37, 38]. In response to this challenge, plants amplify proline production as a countermeasure against the disproportionate presence of Hyp. In the quest for heightened stress resilience, the addition of Hyp to in vitro screenings offers potential. This approach has been harnessed in stress-resistant cell line selection for diverse crops, including peanut [39], *Arabidopsis thaliana* [40, 41], maize [42], and winter oilseed rape [43]. Intriguingly, the application of Hyp-focused screening on banana mutants remains unexplored.

Our study provides a comprehensive analysis of the significant contribution of L-Hyp in the identification of cold-resistant banana mutants, highlighting its impact on the survival rate of rapidly multiplying seedlings. In the course of screening a substantial population subjected to mutagenesis, the deliberate application of L-Hyp, instead of subjecting organisms directly to cold stress, was motivated by the necessity for a more streamlined and precise approach. By concentrating on L-Hyp, we were able to accurately identify mutants exhibiting enhanced resistance to cold environments. The deliberate selection method employed in this study exhibited greater effectiveness when compared to subjecting the entire population to the intricacies of natural cold stress, which is susceptible to logistical difficulties and potential variations in outcomes. This justification harmonizes perfectly with our aim of accurately delineating the desired traits.

### Navigating challenges and leveraging mutagenesis to advance banana breeding strategies

Mutation breeding remains a powerful approach in banana breeding, particularly for enhancing varieties with specific and readily identifiable traits, given the challenges associated with parthenocarpic fruiting and polyploidy [1]. In addressing the existing challenges within the banana industry, such as biotic and abiotic stresses, effective and efficient breeding techniques are crucial for developing new cultivars with superior traits. Notably, mutation breeding presents distinct advantages in this regard. In the context of our study, the successful selection of the *cm784* mutant, characterized by improved cold resistance and other desirable traits, highlights the potential of mutagenesis as a viable strategy. This *cm784* mutant, with its enhanced adaptability to cold stress and other favorable attributes, underscores the promising role of mutation breeding in the creation of novel banana varieties.

Furthermore, our findings indicate that the stomatal activity is reduced in mutants as compared to wildtype, rather than a positive impact on key physiological parameters such as transpiration, intercellular CO_2_ concentration, conductance, and leaf vapor pressure deficit (Fig. 4F). Through our transcriptome analyses, we have also observed a notable enrichment in the plant hormone signaling pathway, highlighting the crucial involvement of plant hormones in regulating stomatal aperture. Significantly, the differential expression of two genes, namely Ma03_g02030 encoding a Tify domain-containing protein and Ma04_g08500 functioning as Jasmonoyl--L-amino acid synthetase GH3.5, has been observed. These findings not only contribute to the advancement of our comprehension regarding the molecular underpinnings of ***cm784***’s heightened cold resistance but also lay the groundwork for forthcoming breeding tactics.

## Conclusion

Our study demonstrates the significant impact of EMS-induced mutagenesis on *Baxijiao* banana seedlings, affecting their growth, development, and cold resistance. We have successfully identified a mutant, cm784, with enhanced cold resistance, which is attributed to heightened antioxidant enzyme activities, osmo-protectant accumulation, and specific molecular mechanisms. The enrichment of genes related to carbohydrate metabolism and secondary metabolite processes suggests the adaptive strategies employed by the mutant to counter cold stress. These findings hold promise for banana breeding and contribute to our understanding of genetic manipulation for enhancing key agronomic traits in this vital crop. Our research provides valuable insights into the potential benefits of mutagenesis for improving banana production and resilience to environmental challenges.

## Materials and methods

### Materials

For the purpose of obtaining sterile multiplication shoots, healthy suckers of *Baxijiao* (*Musa acuminata*, subgroup Cavendish) were sourced from the banana resource conservation nursery at Chinese Academy of Tropical Agricultural Sciences. These healthy suckers were utilized to initiate explants and initiate the process of procuring sterile multiplication shoots.

### EMS mutagenesis treatment

A phosphate buffer solution (pH 5.9, concentration 0.1 mol/L) was prepared by combining NaH2PO4 and Na2HPO4 solutions. The EMS solution with concentrations from 0.0 to 2.0% (0.2, 0.4, 0.6, 1.0, 1.5, 2.0%) was produced using the buffer solution. Modified from Jankowicz-Cieslak and Till [44], sterile banana shoots were mutagenized by immersing them in EMS solutions at 25 °C and 120 rpm for 2–4 hours. After washing, EMS-mutagenized shoots were transferred to fresh Murashige and Skoog medium supplemented with 6-BA (3.0 mg/L). Survival, differentiation, and proliferation of sterilized shoots were quantified over three multiplications.

### Hydroxyproline (L-Hyp) stress screening

After EMS mutagenesis, shoots were subcultured and proliferative buds transferred to EMS-free multiplication medium (M_1_V_1_). To ensure continuity, M_1_V_3_ buds were trimmed. One bud underwent rooting, while another was transferred to L-Hyp supplemented medium. After 25 days, M_1_V_4_ was generated and rooted. To identify desirable mutations, multiplication shoots surviving mutagenesis were transplanted into L-Hyp-supplemented mediums (0.2–1.2 mmol/L). Selection was guided by assay data from survival rates. Unmutagenized shoots (WT) were used as control. Survivability was observed over 25 days for three replication flasks, each containing 30 shoots. Leaves were weighed for fresh weight (FW), lateral buds were removed and transplanted to a rooting medium. After 30 days, root length and stem height were measured. Plants were transplanted to 1:1 coconut husk and vermiculite mix. Phenotypic changes were assessed using Descriptors for Bananas [45].

### Biomass composition assay

To investigate the impact of phenotypic diversity on mutant biomass components, we conducted an analysis of the leaf biomass components of mutants exhibiting phenotypic changes. We measured the biomass components of the third leaf from the top of banana seedlings. The leaves were dried at 50 °C and ground through a 40 mesh screen. Biomass of green leaf tissues was used to measure cell wall as previous study [46]. The total amount of lignin, including acid-soluble and insoluble lignin, was measured as it was in the previous study [47]. All analyses were completed in independent triplicate.

### Evaluation of banana cold tolerance through electrical conductivity assay

To assess banana cold tolerance, we conducted an electrical conductivity assay on banana leaf samples subjected to various cold stress treatments at temperatures of 4, 7, 10, and 13 °C. Fresh leaf segments were immersed in deionized water, and the initial electrical conductivity (EC_initial) was measured after 2 hours. Subsequently, the samples were subjected to the corresponding temperature treatments for 24 hours, followed by thawing at 25 °C for 2 hours, and the final electrical conductivity (EC_final) was measured. The relative electrolyte leakage (REL) was computed using the formula (EC_final/EC_initial), following the previously described method [48]. Higher REL values indicate increased cell membrane damage, reflecting reduced cold tolerance. In addition, a stomatal assay was conducted following the method described previously [49] to determine the size of stomatal guard cells and calculate stomatal density based on the number of stomata per unit of leaf area. The experiment was replicated with multiple biological samples, and statistical analyses were performed to validate the significance of the results.

### Determination of net photosynthetic rate

The Li-6400 portable photosynthesis measurement system (LI-6400 Inc., Lincoln, NE, USA) was employed to determine key parameters, such as net photosynthetic rate. The parameters assessed encompassed Conductance, Intercellular CO_2_ concentration, Transpiration, Leaf Vapor Pressure Deficit, among others. Leaf samples for measurement were procured from the central section of the right side of the third leaf from the plant’s base. Measurements were consistently conducted at 15:00 during clear afternoons.

### Low temperature stress and measurement of physiological and biochemical indices

We sequentially treated mutagenized 45-days acclimated seedlings at 13 °C, 10 °C, 7 °C, and 4 °C for 8 hours each under low temperature stress after the L-Hyp pressure screening, a control group was composed of *Baxijiao* seedling (WT) without EMS/L-Hyp treatment. Physiological indicators such as proline and malondialdehyde (MDA) content, peroxidase (POD) and catalase (CAT) activity were determined. The enzymes CAT, POD, MDA and Proline were extracted from leaves on the same location, For determination, manual instructions (G-Clone® Biotechnology Co., Ltd. Kit)was followed during the specific operation.

### Analysis of banana transcriptome

For RNA extraction, over 500 mg of leaf tissue was collected from the mutant (*cm784*) and wild-type (*Baxijiao*) samples. Total RNA was extracted using the TRIzol reagent (Promega, Beijing, China) following the manufacturer’s protocol. Subsequently, 1 μg of RNA from each sample was used for library preparation using the NEBNext® Ultra™ RNA Library Prep Kit for Illumina® (NEB, Ipswich, MA, USA). The constructed RNA-Seq libraries were subjected to paired-end sequencing with read lengths of 125 bp/150 bp on an Illumina Hiseq 2000 platform (San Diego, CA, USA) at Biomarker Technologies (Peking, China). Differential expression analysis was performed using Fold Change≥2 and FDR < 0.01 as criteria to identify differentially expressed genes (DEGs). GO/KEGG enrichment, GSEA, and protein interaction analyses were conducted for the DEGs.

### Supplementary Information

**Additional file 1.**

## Data Availability

The data and materials underlying this article will be shared on reasonable request to the corresponding author.
