# Supplementary material for: Enhancing cold resistance in Banana (Musa spp.) through EMS-induced mutagenesis, L-Hyp pressure selection: phenotypic alterations, biomass composition, and transcriptomic insights
Source: BMC Plant Biol. 2024 Feb 9;24:101. doi: 10.1186/s12870-024-04775-5 (PMC10854111; doi:10.1186/s12870-024-04775-5)
Supplement: Supplementary file 1 — Additional file 1. [file 12870_2024_4775_MOESM1_ESM.docx]

**Supplemental File**


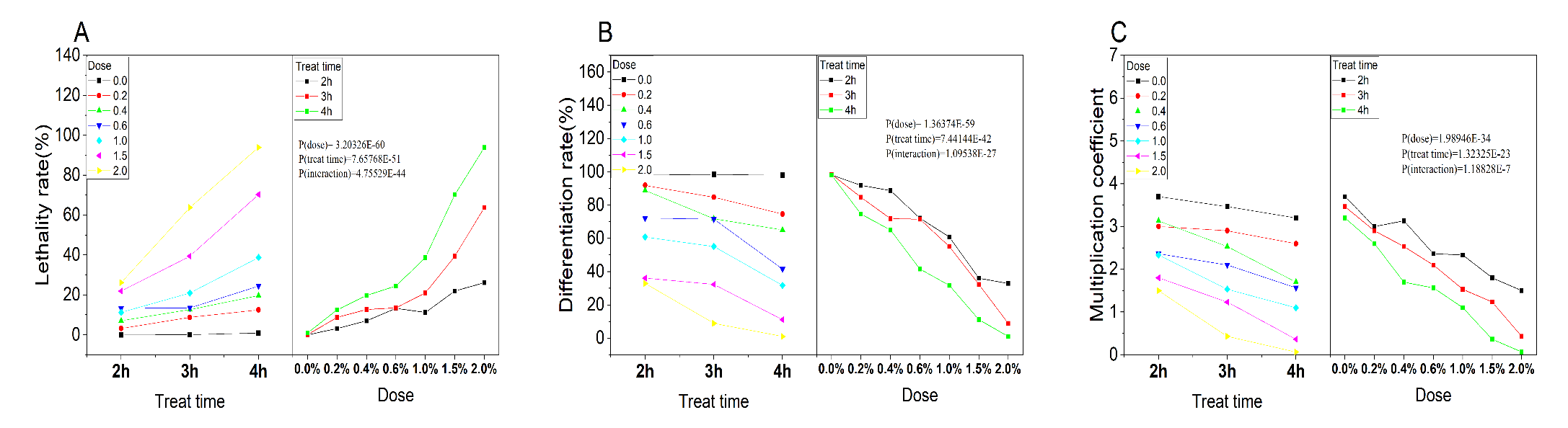


Figure S1. The outcomes of the investigation into the impacts of diverse EMS dosages and treatment durations on lethality rate (A), differentiation rate (B), and multiplication coefficients (C). Post hoc comparisons illustrated that there was a noteworthy difference concerning the interaction of treatment time and dosage (P < 0.0001) in terms of plant response variables. The values are shown as means ± standard deviation. Two-way ANOVA was conducted, and the results revealed significant P values for the dose, treatment time, and interaction variables. The data presented herein are the mean ± standard deviation. The experiments were executed independently at least thrice.


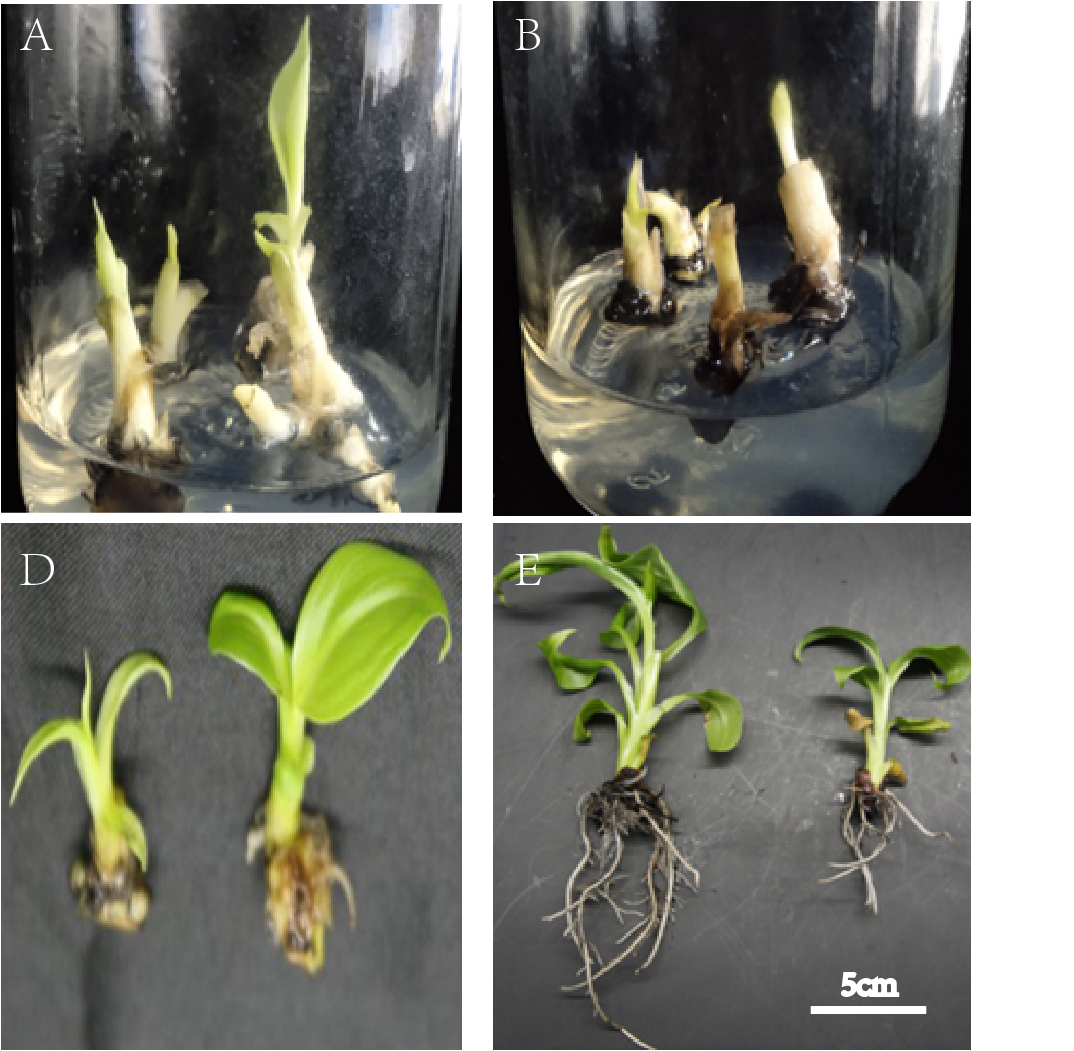


Figure S2. The effect of EMS mutagenesis on the induction of rooting in banana tissue culture plantlets. A. Growth state of control plantlets after 1 week. B. Growth state of plantlets treated with EMS after 1 week. C. Growth state of plantlets in vitro after 2 weeks (left: EMS-treated banana plantlets, right: control plantlets). D. Rooting state after 1 month (left: control, right: EMS-treated tissue culture plantlets). Scale bar represents 5 cm.


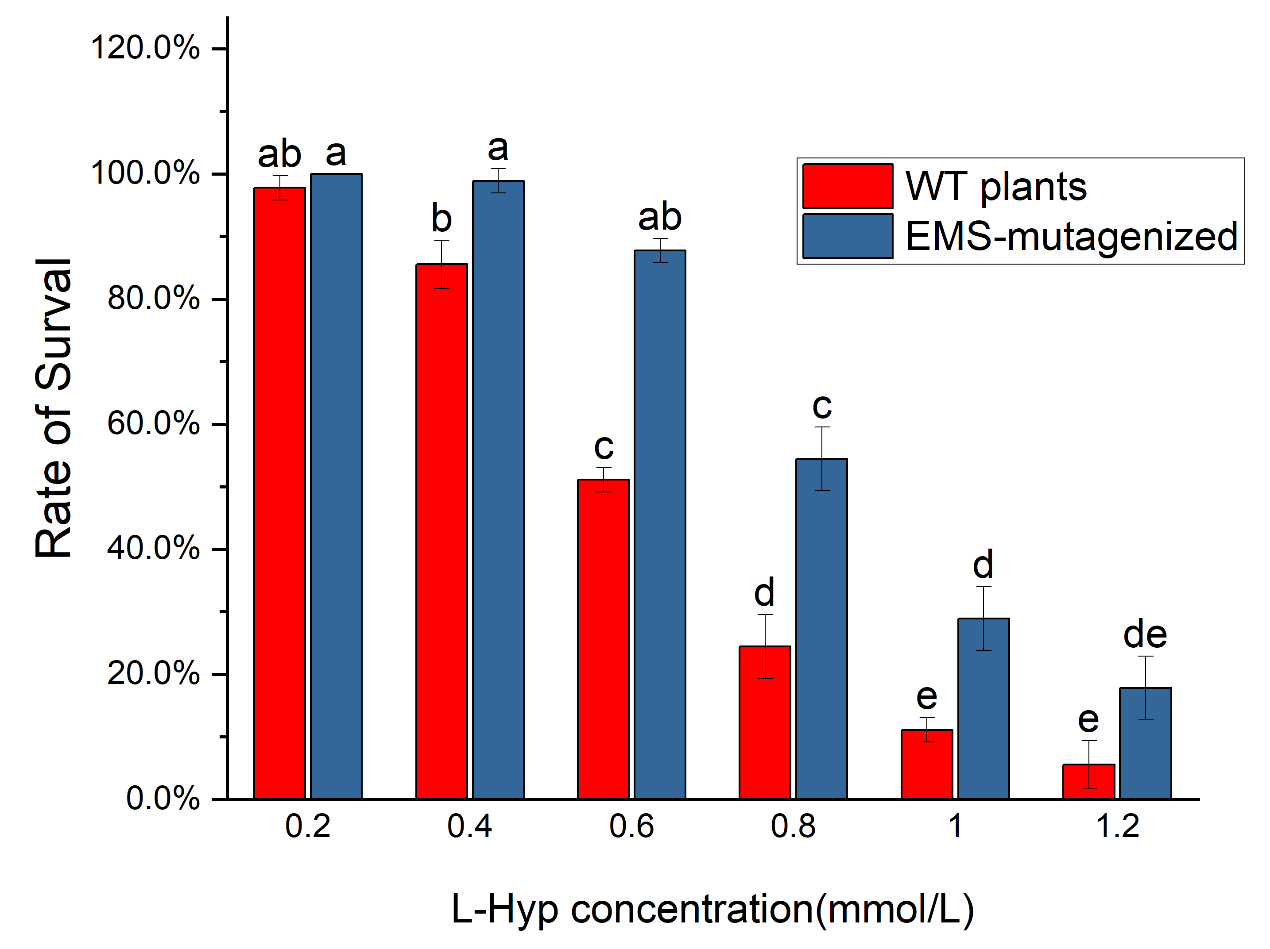


Figure S3. Effect of L-Hyp on the survival rate of banana tissue culture plantlets. Means represented by the same letter do not show significant differences at P ≤ 0.05 based on Turkey's test.


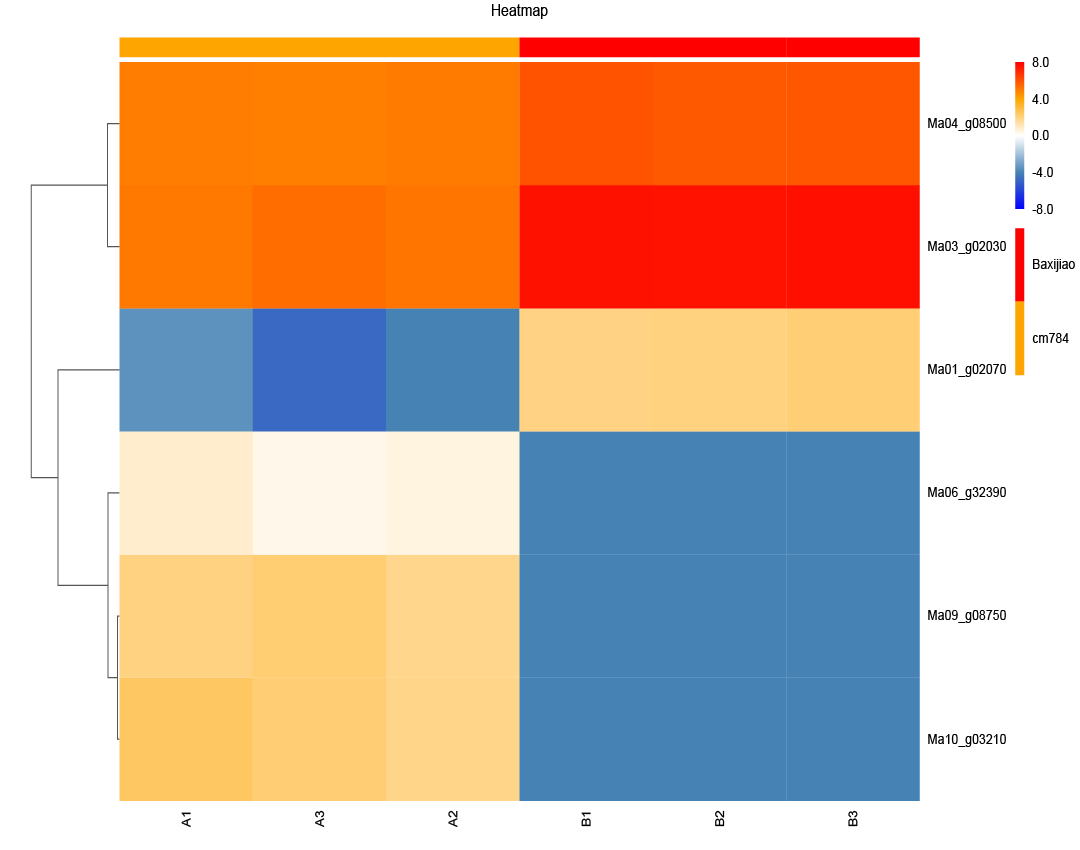


Figure S4 Heatmap illustrating the FPKM gene expression levels of *cm784* and *Baxijiao* underlining the differential expression patterns.
